# Prognosis of rectal cancer patients improves with downstaging by intensified neoadjuvant radiochemotherapy - a matched pair analysis

**DOI:** 10.1186/1471-2407-13-388

**Published:** 2013-08-16

**Authors:** Leif Schiffmann, Gunther Klautke, Nicole Wedermann, Michael Gock, Friedrich Prall, Rainer Fietkau, Bettina Rau, Ernst Klar

**Affiliations:** 1 Department of General, Thoracic, Vascular and Transplantation Surgery, University of Rostock, Schillingallee 35, Rostock 18057, Germany; 2 Department of Radiotherapy, Sozialstiftung Bamberg, Buger Str. 80, Bamberg 96049, Germany; 3 Institute of Pathology, University of Rostock, Strempelstr. 14, Rostock 18055, Germany; 4 Department of Radiotherapy, University of Erlangen, Universitätsstr. 27, Erlangen 91054, Germany

**Keywords:** Rectal cancer, Intensified neoadjuvant radiochemotherapy, Cancer related survival, Disease free survival

## Abstract

**Background:**

Neoadjuvant radiochemotherapy has been proven superior to adjuvant treatment in reducing the rate of local recurrence without impairing cancer related survival or the incidence of distant metastases in standard protocols of neoadjuvant radiochemotherapy. The present study aimed at addressing the effects of an intensified neoadjuvant radiochemotherapy on long term cancer related and disease free survival.

**Methods:**

A total of 387 patients underwent oncologic resection for rectal cancer in our institution between January 2000 and December 2009. There were 106 patients (27.4%) who received an intensified radiochemotherapy protocol completely and without excluding criteria (study group). A matched pair analysis was performed by comparing the study group with patients undergoing primary surgery and postoperative radiochemotherapy, if necessary and possible (control group). Matching was carried out in descending order for UICC stage, R-status, tumor height, T-, N-, V-, L-, M- and G-category of the TNM-system according to the histopathological staging. Follow-up data included local recurrence rate, cancer related and disease free survival.

**Results:**

In the study group histopathological work-up of the specimen revealed a treatment response in terms of tumor regression in 92.5% (98/106) of these patients. Undergoing intensified neoadjuvant RCT the actuarial cancer related and disease free survival was 67.9% and 70.4%, local recurrence was 5.7% after an observation period of 4.3 ± 2.55 years. In the control group cancer related and disease free survival was 71.7% and 82.7%, local recurrence was 4.7% after an observation period of 3.8 ± 3.05 years revealing no statistical significant difference between the two groups. Moreover, estimated 5-year results of cancer related survival (66.7% vs 67.9% (controls)), the disease free survival (66.7% vs 79.9% (controls)) as well as subgroup analysis of UICC 0-III and UICC IV patients showed no difference between the study and control group as well.

**Conclusion:**

In our study, intensified neoadjuvant radio-chemotherapy shows a high rate of tumor regression. The resulting inferior histopathological tumor stage shows the same long term local control and systemic tumor control as the control group with a primary more favorable tumor stage.

## Background

For advanced rectal cancer, neoadjuvant radiochemotherapy (RCT) has been proven to reduce the rate of local recurrence in comparison to postoperative treatment [1] or preoperative radiotherapy alone [2,3]. German guidelines state exact treatment rules for UICC stage I to III and localization of cancer in the rectum [4] depending on the local tumor stage at initial tumor diagnosis. Since there is no benefit of neoadjuvant radio-(chemo)therapy on cancer related survival or distant metastases [1-3], efforts have been made to improve the systemic results of these protocols. By adding a second drug to the neoadjuvant radiochemotherapy, the rate of complete responses and tumor regression grade could be increased [5-7]. A complete response has been shown to be a predictive marker for disease free and cancer related survival. Thus, an intensified neoadjuvant RCT protocol was introduced at several institutions including irinotecan or oxaliplatin [7-18] which uniformly confirmed improved tumor response resulting in complete response rates up to 23% and tumor/nodal downstaging in up to 65% [19]. However, long-term follow-up data such as overall or disease free survival as well as local recurrences of any of the intensified neoadjuvant radiochemotherapy protocols are still not available.

The aim of our study was therefore to investigate, whether an intensified neoadjuvant radiochemotherapy leads to an improvement of the respective oncological long term end-points beyond downstaging and tumor regression compared with patients receiving no neoadjuvant treatment at all.

## Patients and methods

A total of 387 patients with rectal cancer underwent oncologic resection in our institution between January 2000 and December 2009 and were included into this retrospective matched pair analysis by screening the pathological data base. The term rectum carcinoma was applied to adenocarcinomas located at a distance from 0 to 16 cm from the anal verge measured by rectoscopy. The cancer was located in either the lower (0- < 6 cm), middle (6- < 12 cm) or upper (12-16 cm) rectum.

According to the current German guidelines, indication for neoadjuvant RCT was given for T3, T4 and/or nodal positive tumors of the lower and middle third of the rectum. In the upper third of the rectum, the only indication for neoadjuvant treatment was a T4 cancer.

Pretherapeutic studies included routine laboratory analysis, ECG, endoscopy and biopsy, abdomen ultrasound or computertomography, endoluminal ultrasound for local staging and chest radiography.

In our institution, patients usually received an intensified radiochemotherapy with some modification of the chemotherapy drugs during the observation period. From January 2000 to January 2002 patients received a combination of a continuous infusion 5-FU (250 mg/m2 per day) over 31 days, irinotecan (initially 6 times, once a week with 40 mg/m2; later 4 times, once weekly in week 1, 2, 4, and 5 with 60 mg/m2) and a local radiation five days a week with a single dose of 1.8 Gy adding up to 50.4 Gy (last three doses were reduced). From February 2002 5-FU was replaced by a daily intake of Capecitabine with a single dose between 1000 and 1650 mg/m2. Doses of radiation were no longer reduced and reached a cumulative dose of 55.4 Gy. Oxaliplatin had been applied instead of Irinotecan in eight patients. Following surgery, patients usually received adjuvant chemotherapy with 5-FU with or without folinic acid or Capecitabine according to the recommendations of the German Cancer Society (DKG). Additionally, irinotecan or oxaliplatin was applied in 23 patients.

The type of surgery depended on localization of the tumor, preoperative stool incontinence and general condition of the patient. Generally, patients received a total mesorectal excision (TME) for all cancers located between 0 and 12 cm [20,21] and a partial mesorectal excision (PME) for all cancers located higher than 12 cm. All anastomoses were performed by double stapling technique.

After identifying all patients with a rectal adenocarcinoma, we eliminated all patients receiving a short term radiation (5x5 Gy), conventional neoadjuvant radiochemotherapy and all patients having complications during the intensified neoadjuvant treatment or having another malignancy in their history. Thereafter, we stratified all remaining patients who received an intensified neoadjuvant RCT as the “study group”, whereas patients without any neoadjuvant treatment before surgery represented the “control group”. There were 106 patients eligible to the study group, another 106 patients of the control group were matched in decreasing preference by UICC stage, R-Status, tumor height, T-, N-, V-, L-, M- and G-category of the TNM-system. Tumor stage (TNM/UICC) was based exclusively on histopathology of the surgical specimen and operative findings. All patients with non-resected residual metastatic disease (hepatic and/or pulmonary metastases) were classified as R2-resections.

Histopathological work-up generally included a statement of tumor response to the neoadjuvant radiochemotherapy protocol for all neoadjuvant treated patients. Regression was divided into four categories: no response – only vital tumor was seen; poor to moderate regression – large vital tumor complexes were seen in the majority of the blocks. Good regression – better than category poor to moderate response but not a complete response; complete response – no residual tumor was found. Further pathological subclassification in low- moderate – strong tumor regression as previously reported [5] was not routinely performed.

Adjuvant treatment generally consists of a combination of a continuous infusion 5-FU (1000 mg/m2 per day) and a local radiation five days a week with a single dose of 1.8 Gy and a 5.4 Gy boost adding up to 55.8 Gy. After completion of radiation, a chemotherapy analog to the postoperative chemotherapy of the study group was administrated.

Follow-up information was obtained from all patients of the study and control group in 2011. The mean interval between oncologic resection and follow-up was 4.3 ± 2.55 years in the study group and 3.8 ± 3.05 years in the control group. On the basis of a standardized questionnaire primary end-points were analyzed and included local recurrence, distant metastases, overall survival and cancer related survival. Follow-up examinations were carried out in cooperation with the referring physicians according to the German S3 guidelines for colorectal cancer [4] and provided a comprehensive and complete data collection.

The study was approved by the Medical Ethical Committee of Rostock University.

### Statistical analysis

Statistical analysis was performed using Statistical Package for Social Science (SPSS) version 15.0. Statistical analysis was done using Pearson’s chi-square test (Fisher’s exact test). Survival curves were calculated according to the Kaplan-Meier method. Survival curves were tested for significant differences using the log-rank test. A p value of < 0.05 was considered as statistically significant.

## Results

106 patients receiving an intensified neoadjuvant RCT (treatment group) were matched with 106 patients, who did not receive any neoadjuvant treatment (control group), on the base of histopathological findings, for UICC stage, R-Status, tumor height, T-, N-, V-, L-, M- and G-category of the TNM-system.

Histopathology revealed any kind of local tumor regression in 92.5% of patients after intensified neoadjuvant radiochemotherapy. In this patients’ subset, a poor to moderate response was found in 45.3%, a good response 39.5% and a complete response in 7.5% (ypT0N0). At a minimum, downstaging was achieved in about 30% of all cases (Table 1: UICC-stage 0 and I).

**Table 1:** Cancer related characteristics in the study and control groups

	**All patients % (n = 212)**	**Study group with neoadjuvant treatment % (n = 106)**	**Control group without neoadjuvant treatment % (n = 106)**	**p-value**
Infiltration (pT)				0.02
yT0	4.2	8.5	0	
(y) T1	7.5	5.7	9.8	
(y)T2	28.8	27.4	30.2	
(y)T3	53.3	54.7	51.9	
(y)T4	6.1	3.8	8.5	
Lymph node metastasis (pN)				0.61
Number of nodes examined	17.2	15.3	19.1	<0.001
(y)N0	51.4	52.8	50.0	
(y)N1	25.9	27.4	24.5	
(y)N2	22.6	19.8	25.5	
UICC stage				0.31
UICC 0	1.9	3.8	0	
UICC I	27.4	24.5	30.2	
UICC II	16.0	17.0	15.1	
UICC III	31.1	31.1	31.1	
UICC IV	23.6	23.6	23.6	
R-Category (incl. UICC IV)				0.59
R0	76.8	79.0	74.5	
R1	5.2	5.7	4.7	
R2	18.0	15.2	20.8	
V-Category				<0.01
V0	71.1	81.0	61.3	
V1	23.7	15.2	32.1	
V2	5.2	3.8	6.6	
L-Category				0.08
L0	80.3	85.4	75.2	
L1	19.7	14.6	24.8	
M-Category				1.00
M0	77.4	77.4	77.4	
M1	22.6	22.6	22.6	
G-Category				0.15
G1	11.4	15.2	7.5	
G2	72.0	66.7	77.4	
G3	16.6	18.1	15.1	
Response to neoadjuvant therapy				
No response		7.5		
Poor to moderate regression		45.3		
Good regression		39.6		
Complete regression		7.5		
Adjuvant postoperative Radio- chemotherapy (% and patients)			52.9 (54 pts.)	
Completing Chemotherapy after surgery (% and patients)		59.4 (60)		
Localization				0.70
Upper Rectum	4.8	3.8	5.7	
Middle Rectum	41.9	40.4	43.4	
Lower Rectum	53.3	55.8	50.9	
Tumorheight (cm)	5.6	5.3	5.9	0.22

Tables 1 and 2 show the results of the matching. Matching was well balanced for UICC, presence of positive lymph nodes, R-status, presence of distant metastases and tumor localization between the two groups. In contrast, significant differences were observed concerning a less advanced T-stage (p = 0.02), a lower number of lymph nodes examined (p < 0.001), a lower percentage of vascular invasion (p < 0.01), and a lower comorbidity in the study group. Nine patients (8.5%) revealed a complete local response corresponding by ypT0. In UICC stage 0-III patients, one patient of each group had a local R2 resection, 5 patients of the treatment group and seven patients of the control group had a local R1 resection.

**Table 2:** Biological data and comorbidity in the study and control groups

	**All patients % (n = 212)**	**Study group with neoadjuvant treatment % (n = 106)**	**Control group without neoadjuvant treatment % (n = 106)**	**p-value**
Gender ratio (f : m)	1 : 1.94	1 : 2.92	1 : 1.35	<0.01
Age (mean)	65.4	62.3	68.5	0.06
Comorbidity	77.0	68.6	85.5	<0.01
Pulmonary	9.1	5.7	12.5	0.01
Cardiovascular	23.9	21.9	26.0	0.52
Renal	7.2	6.7	7.7	0.80
Diabetes	19.1	11.4	26.9	<0.01
Hypertension	47.8	40.0	55.8	0.03
Others	48.9	39.0	58.7	<0.01
ASA score (mean)	2.4	2.3	2.5	0.05

**Figure 1:**
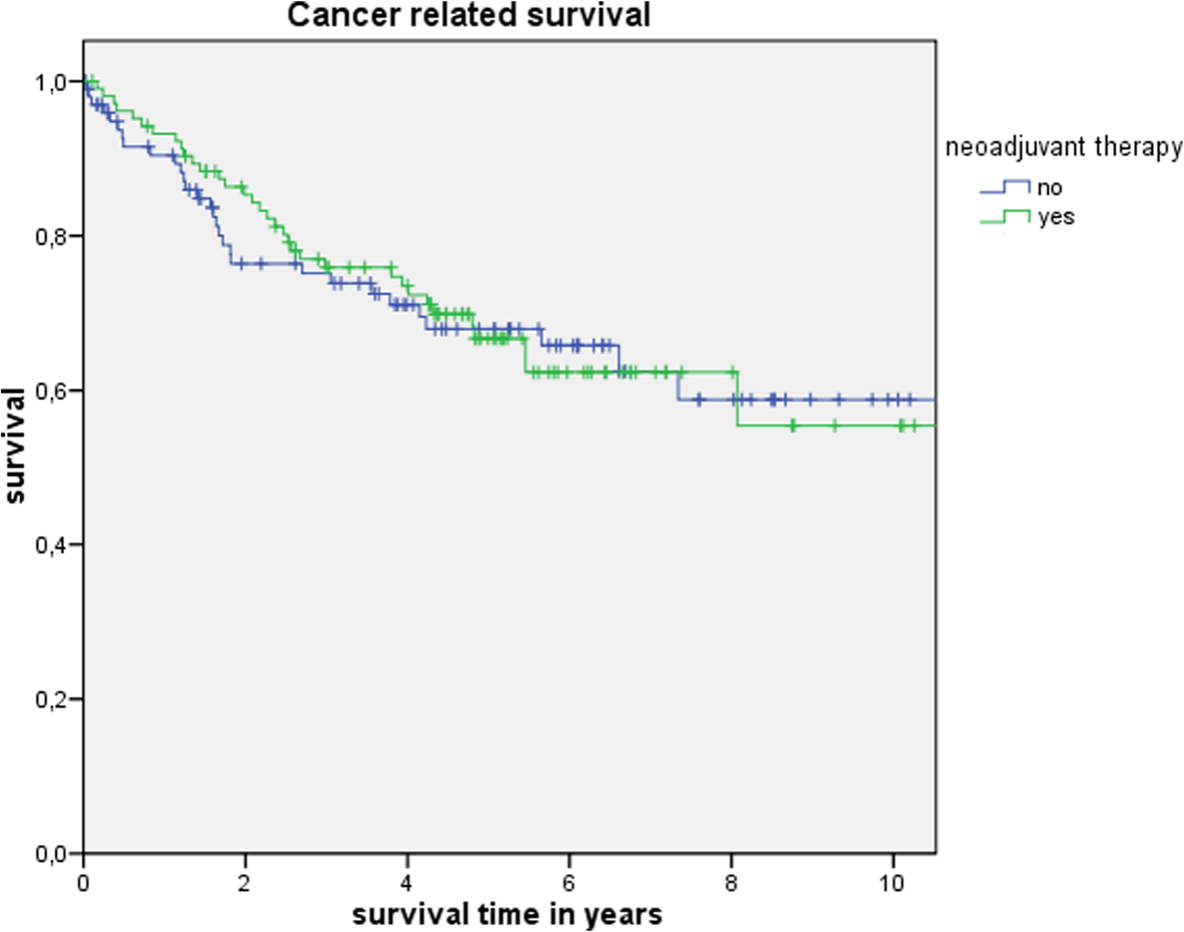
This figure shows no difference in cancer related survival UICC stage 0 to IV depending on administrating intensified neoadjuvant radiochemotherapy (p = 0.88) based on the matched pair analysis described in the text.

**Figure 2:**
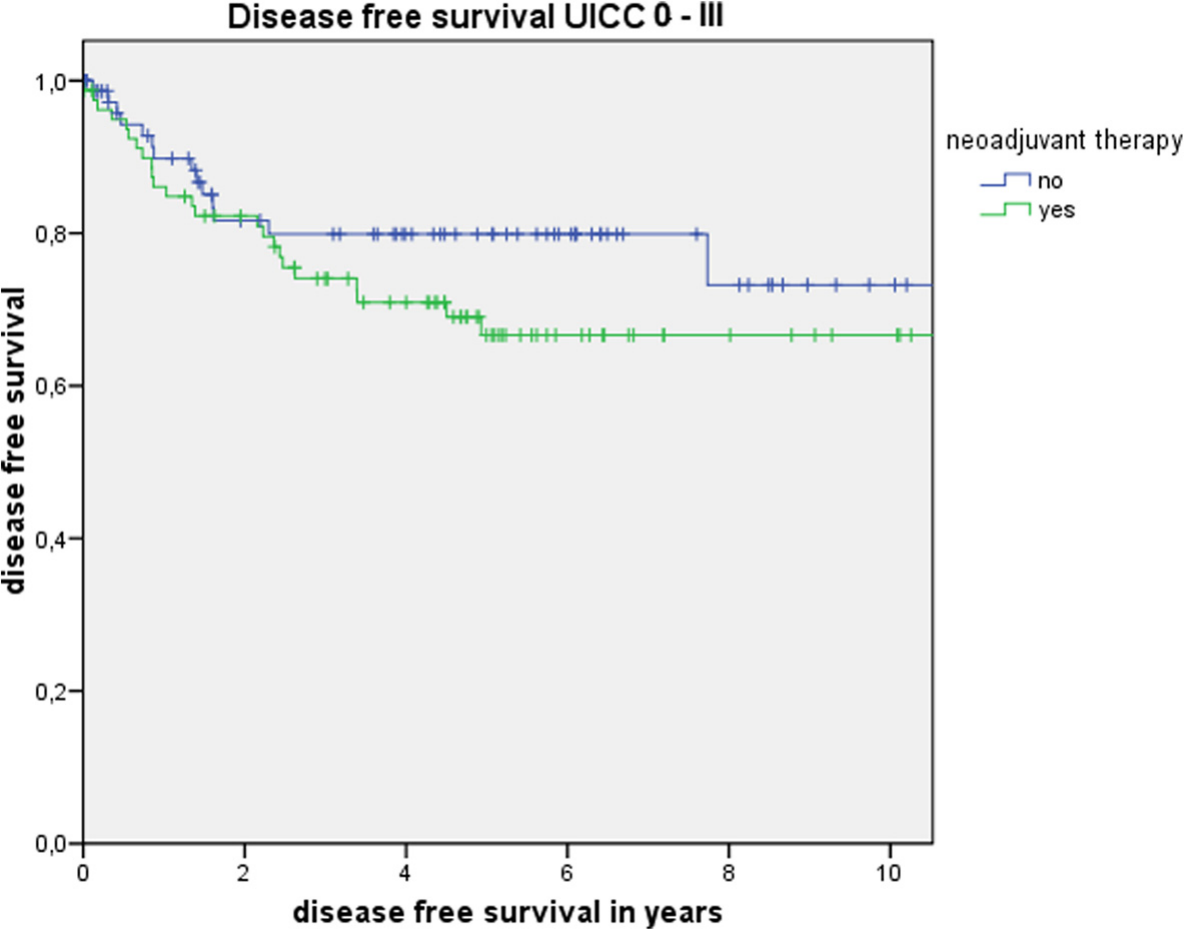
This figure shows no difference in disease free survival UICC stage 0 to III depending on administrating intensified neoadjuvant radiochemotherapy (p = 0.22) based on the matched pair analysis described in the text.

**Figure 3:**
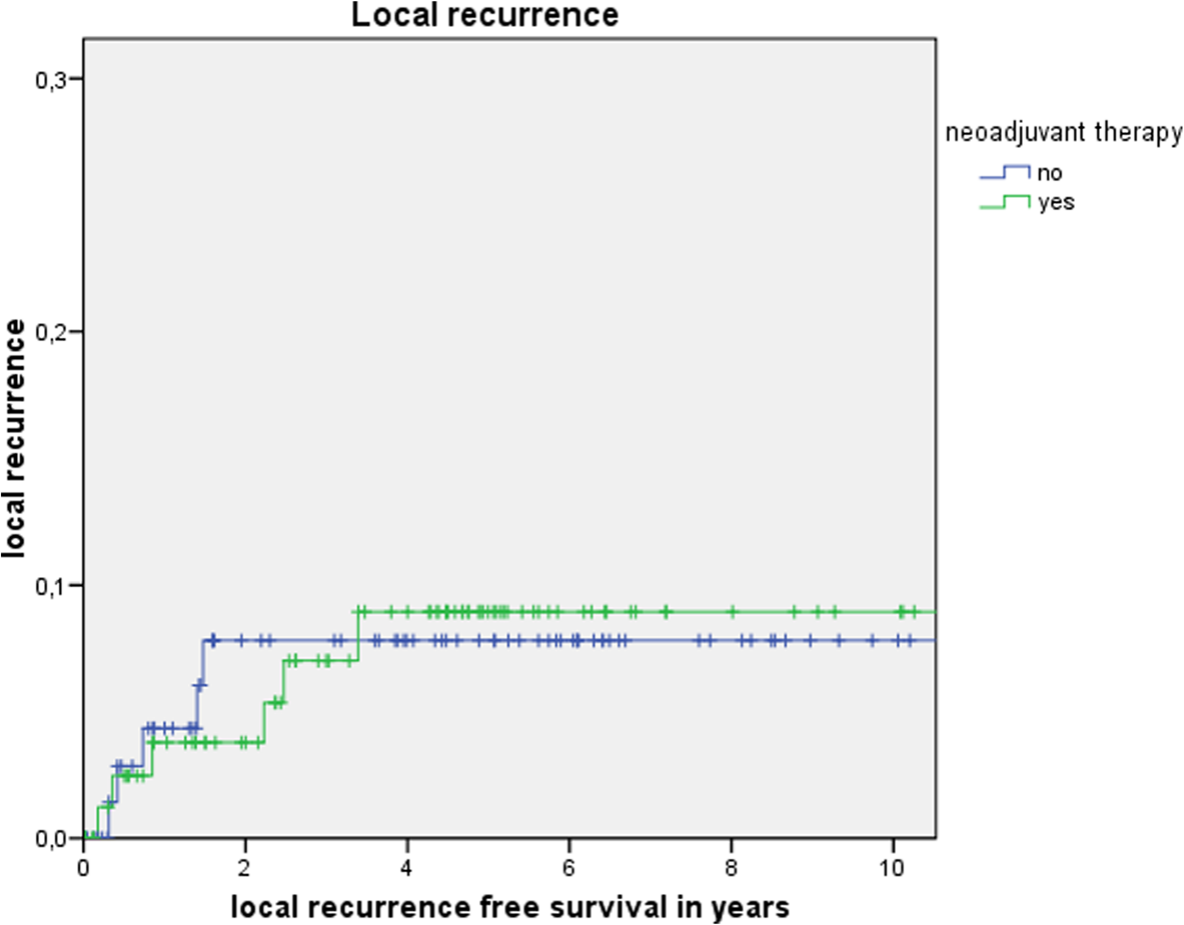
This figure shows no difference for local recurrence depending on administrating intensified neoadjuvant radiochemotherapy (p = 0.98) based on the matched pair analysis described in the text.

**Figure 4:**
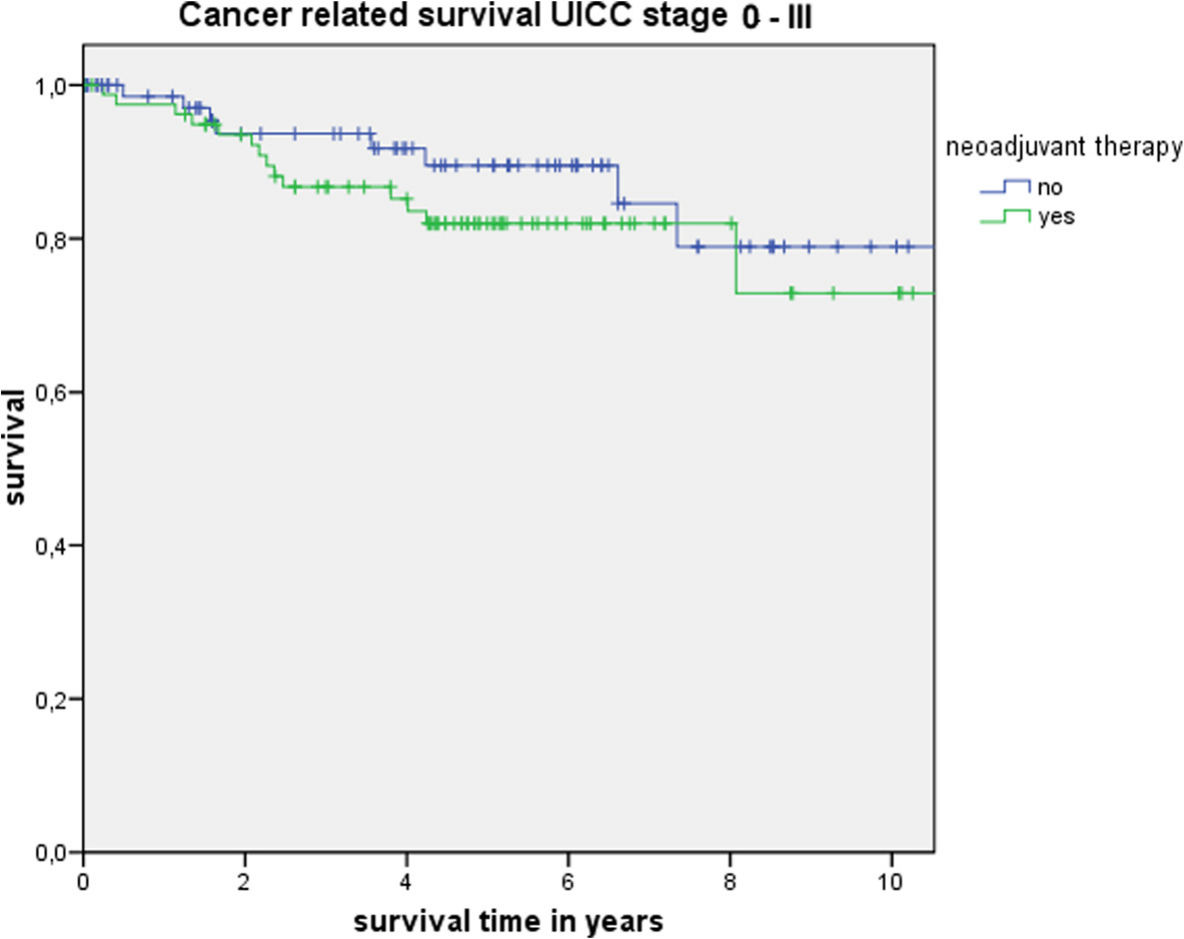
This figure shows no difference in cancer related survival UICC stage 0 to III depending on administrating intensified neoadjuvant radiochemotherapy (p = 0.32) based on the matched pair analysis described in the text.

**Figure 5:**
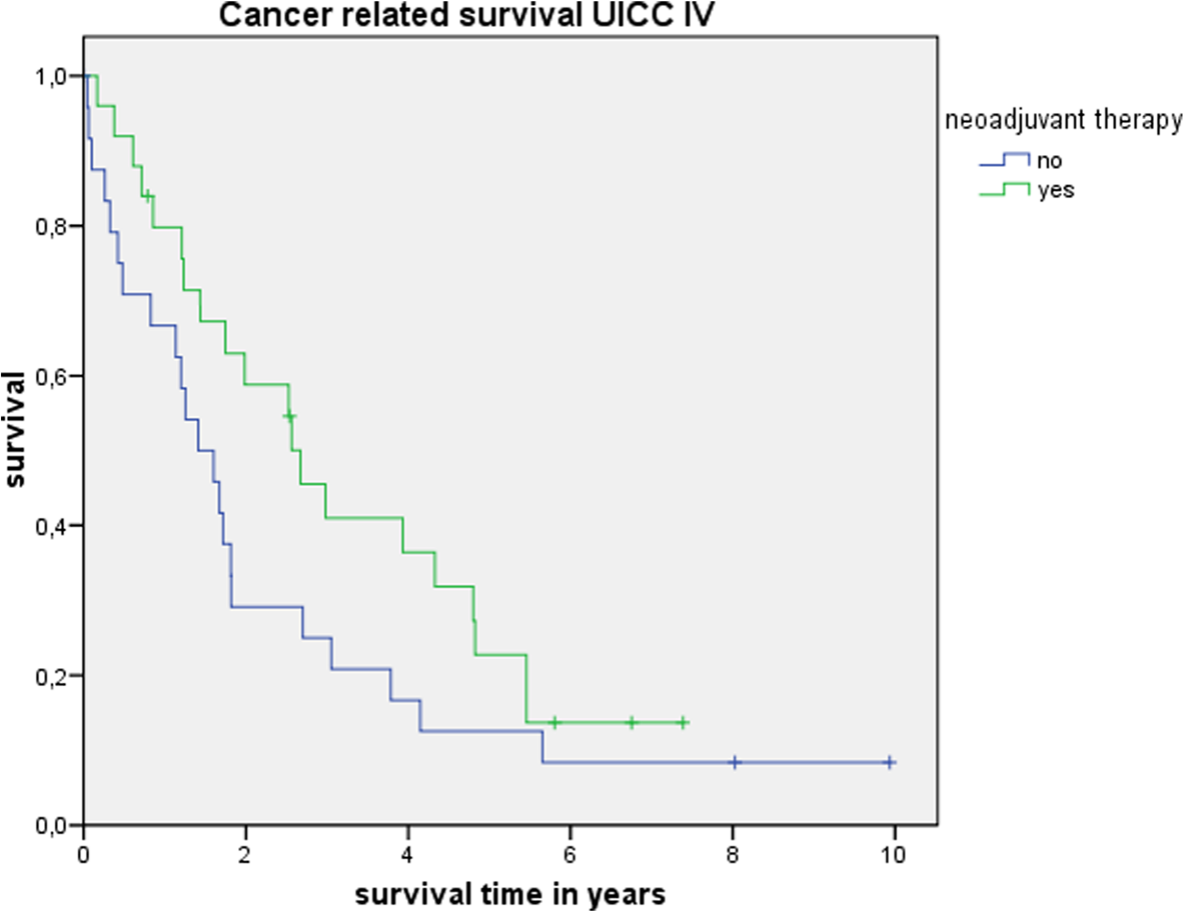
This figure shows no difference in cancer related survival UICC stage IV depending on administrating intensified neoadjuvant radiochemotherapy (p = 0.11) based on the matched pair analysis described in the text.

**Figure 6:**
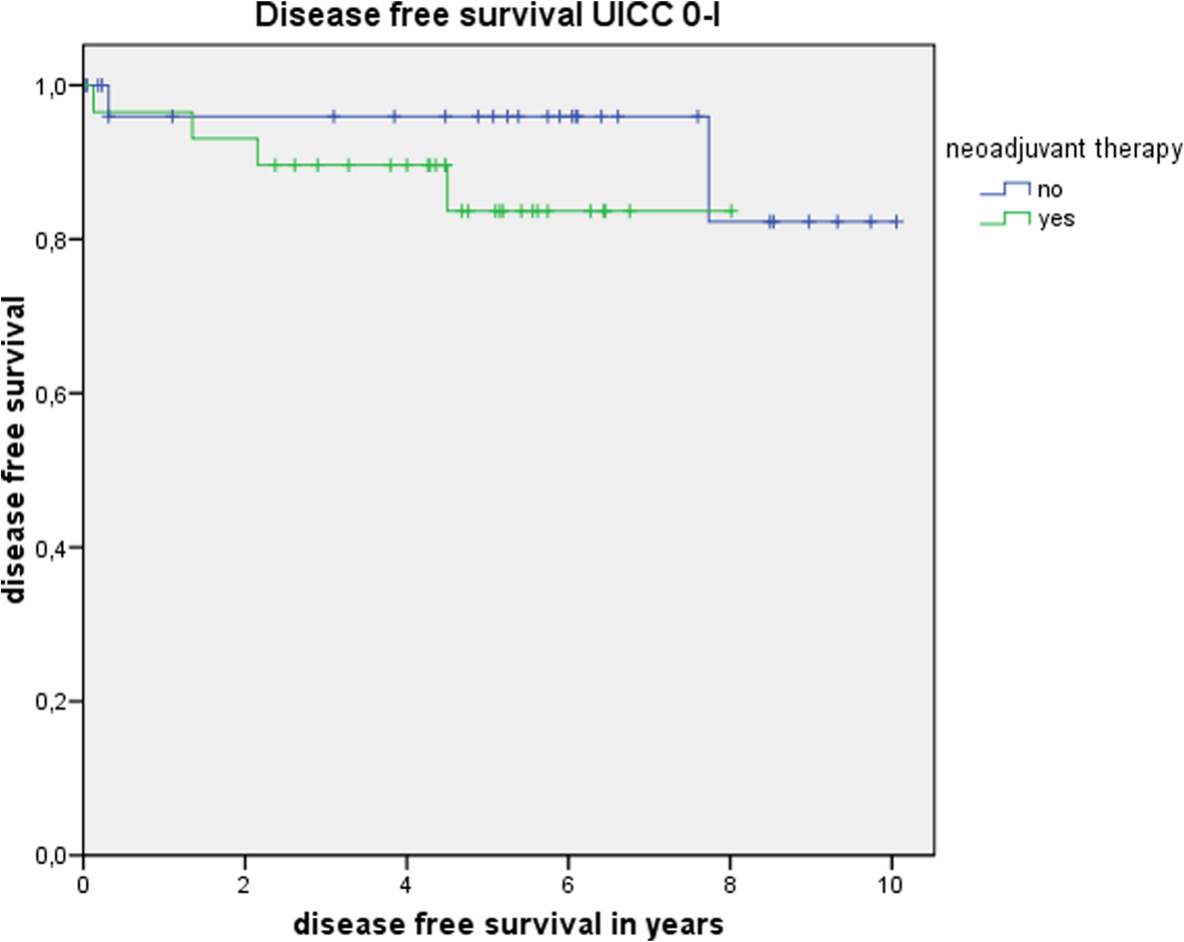
This figure shows no difference in disease free survival UICC stage 0-I depending on administrating intensified neoadjuvant radiochemotherapy (p = 0.27) based on the matched pair analysis described in the text.

Table 3 shows the actuarial and estimated 5-year survival data showing no significant differences. Figure 1 shows the overall estimated cancer related survival, Figure 2 the estimated disease free survival and Figure 3 the local recurrence rates in the study and control group in all patients without showing any differences between groups. If only patients with UICC stage 0-III (Figure 4) versus UICC stage IV (Figure 5) and UICC stage 0-I (Figure 6) are separately analyzed, cancer related and disease free survival are not different between the two groups as well. In subgroup analysis, R0 resected, N1 and/or N2 positive patients showed no significant differences in cancer related survival. In behave of low patient numbers, these figures are not shown. However, UICC-stage dependent survival benefits of the UICC 0-III (Figure 4) versus UICC stage IV (Figure 5) become apparent.

**Table 3:** Actuarial and estimated cancer-related outcome in the study and control group

	**All patients % (n = 212)**	**Study group with neoadjuvant treatment % (n = 106)**	**Control group without neoadjuvant treatment % (n = 106)**	**p-value**
Follow-Up in years (mean)	4.04	4.3 ± 2.55	3.8 ± 3.05	0.172
**Actuarial outcome**				
Cancer related survival				
All UICC stages	69.8	67.9	71.7	0.65
UICC stage 0-III	86.4	82.7	90.1	0.25
Disease free survival				
UICC stage 0-III	76.5	70.4	82.7	0.09
Local recurrence				
All UICC stages	5.2	5.7	4.7	1.00
UICC stage 0-III	6.8	7.4	6.2	1.00
**Estimated 5-year outcome**				
Cancer related survival				
All UICC stages	67.3	66.7	67.9	0.88
UICC stage 0-III	85.8	82.0	89.6	0.32
Disease free survival				
UICC stage 0-III	73.3	66.7	79.9	0.22
Local recurrence				
All UICC stages	8.4	9.0	7.8	0.98
UICC stage 0-III	8.5	9.2	7.9	0.95

## Discussion

In our study we retrospectively analyzed the long term results of patients with rectal cancer subjected to intensified neoadjuvant RCT compared with patients receiving no neoadjuvant treatment. In the absence of reliable long-term outcome data of intensified treatment protocols we report for the first time on cancer-related and disease-free survival as well as local recurrence after an overall observation period of about 4 years after oncologic resection.

The main finding of our study is that intensified neoadjuvant RCT resulted in a high tumor regression and that patients’ long term prognosis after intensified RCT matches prognosis of patients with the same postoperative histopathological staging without neoadjuvant RCT. In consequence, prognosis correlates with downstaging; e.g. a patient’s prognosis with an ypT1N0M0 cancer after downstaging is as good as the prognosis of a pT1N0M0 patient. Since our patient cohort was recruited over an observation period of 10 years with considerable changes in pretherapeutic diagnostic staging approaches, meaningful evaluation of downstaging was not possible, which is one of the drawbacks of our study. However, in a previous report, we tried to evaluate the effect of the intensified neoadjuvant treatment regimen by histology on the basis of tumor fibrosis [5].

The tumor regression and complete remission are prognostic factors for overall survival [22,23]. The new German Rectal Cancer Trial show a higher rate of complete remission without a significantly higher rate of toxicity by integrating a second drug in the neoadjuvant radiochemotherapy schedule [24].

A further prognostic factor is to achieve an ypN0-category. Looking at patients with UICC-stage II and III, without neoadjuvant therapy, the survival data differ significantly. If, by the way of neoadjuvant therapy a downstaging from a cN + category to a ypN0-category has been reached, and the prognosis is as good as in patients with a primary pN0-category, it would be an excellent result for intensified neoadjuvant radiochemotherapy. But this is exactly, what our matched pair analysis shows. In terms of local control and survival there are no statistically significant differences in the reached ypTNM-category or in the primary pTNM-category.

One critical point in neoadjuvant treatment is the risk of overstaging in the preoperative situation. In the multicenter German Rectal Cancer trial [1] the risk was 18%, but there is also a risk of understaging. A degree of uncertainty despite better diagnostics must be accepted.

In the present analysis we matched also according to the resection status, knowing that neoadjuvant radiochemotherapy reduces the risk of incomplete resection and also knowing, complete resection is an important prognostic factor for local control and survival. This is perhaps one of the reasons that we see no benefit from neoadjuvant radiochemotherapy in local control, as seen in a couple of randomized trials like the German Rectal Cancer Trial or the MRC-Trial before [1,25]. In our control group the rate of local recurrence of 5.2% after an average follow-up of 4 years is rather low compared to other authors describing 13% after 5 years [1,26]. This may be attributed to a high surgical quality standard in performing TME in our department, and a high standard of radiooncological quality by a consequent postoperative radiochemotherapy if necessary, with a high density of the radiation dose, which is also a prognostic factor for local control [27].

The cancer related survival in UICC stage 0-III is 82.7% in the study group in comparison with 76% in the neoadjuvant treatment arm of the German Rectal Cancer Trial [1].

Comparable results to our findings in rectal cancer can be observed in the neoadjuvant therapy of other gastrointestinal tumors. Patients with primarily inoperable or borderline resectable tumors and an UICC stage III category, receiving neoadjuvant radiochemotherapy, following complete resection, have the same prognosis as patients with a primarily resectable tumor situation and an UICC stage I/II [28]. Other examples are adenocarcinoma of the esophagus and the esophagogastric junction. The POET –Trial [29] shows a survival benefit for patients with an ypN0 category in comparison with an ypN + category.

Up to now, there are four randomized trials in rectal cancer, comparing neoadjuvant radiochemotherapy to intensified neoadjuvant radiochemotherapy. The follow up period is too short for long-term results in local control and survival.

## Conclusions

Therefore our matched pair analysis is one of the first steps to suggest, that intensified neoadjuvant radiochemotherapy improves survival in patients with rectal cancer by downstaging to a better tumor situation.

## Competing interests

The authors declare that they have no conflicts of interest.

## Authors’ contributions

LS, GK, NW and MG conceived and coordinated the study, collected patients’ data and participated in the statistical analysis. LS drafted the manuscript. FP, RF, BR and EK participated in preparing and drafting the manuscript. All authors read and approved the final manuscript.

## Pre-publication history

The pre-publication history for this paper can be accessed here:

http://www.biomedcentral.com/1471-2407/13/388/prepub
